# Usefulness of noninvasive diagnostic procedures for assessment of methotrexate hepatotoxicity in patients with rheumatoid arthritis

**DOI:** 10.1007/s00296-021-05059-z

**Published:** 2021-12-06

**Authors:** Marek Frankowski, Jerzy Świerkot, Marek Gomułkiewicz, Lucyna Korman, Marta Skoczyńska, Aleksandra Starba

**Affiliations:** 1 Department of Rheumatology and Internal Medicine, Wroclaw Medical University, Wroclaw, Poland; 2 Department of General Radiology, Interventional Radiology and Neuroradiology, University Clinical Hospital, Wroclaw, Poland; 3 Clinic of Rheumatology and Internal Medicine, University Clinical Hospital, Wroclaw, Poland

**Keywords:** Methotrexate, Liver fibrosis, Rheumatoid arthritis, Elastography, PIIINP

## Abstract

Methotrexate (MTX) is recommended as a first-line treatment for rheumatoid arthritis (RA). There are no strict guidelines regarding monitoring for liver damage in RA patients. This study aimed to evaluate noninvasive diagnostic procedures in assessing liver fibrosis in RA patients. Ninety-six RA patients were recruited for this study. The procollagen III N-terminal peptide (PIIINP) serum level was measured in all patients. The Enhanced Liver Fibrosis score (ELF-1) was calculated for 82 patients. Transient elastography (TE) was performed in 91 patients, those examined were divided into two groups: a study and control group, comprising patients with and without risk factors for liver fibrosis, respectively. The TE result correlated only with the body mass index—BMI (*p* < 0.05); there was no correlation with the cumulative MTX dose (*p* = 0.33). The TE result was significantly higher in those with risk factors for liver fibrosis than in those without risk factors (TE result >  = 7.1 kPa 28/42 vs 13/41, HR = 2.103, Mann–Whitney *U* test, approximately 0.02). There was a positive correlation between the PIIINP level and body weight (*p* = 0.028), cumulative MTX dose (*p* = 0.007), RA activity (*p* = 0.028) and diabetes mellitus (DM) (*p* = 0.001). There was a positive correlation between the ELF-1 score and age (*p* < 0.001), cumulative MTX dose (*p* = 0.007) and RA activity (*p* < 0.001). The PIIINP level and ELF-1 score are not organ specific, and readings may vary depending on RA activity. TE is organ specific and can be performed by a skilled ultrasonographer might be useful to assess actual liver condition.

**Supplementary Information:**

The online version contains supplementary material available at 10.1007/s00296-021-05059-z.

## Introduction

Methotrexate (MTX) is an essential disease-modifying antirheumatic drug (DMARD) used in rheumatoid arthritis (RA) therapy due to its efficacy, relatively good tolerance and low price-to-efficacy ratio [1, 2]. The most common side effects are gastrointestinal ailments and hepatotoxicity (usually manifesting as elevated aminotransferase activity). The long-term administration of MTX may cause liver fibrosis; however, recent studies have suggested that patients with risk factors might be more important [3, 4].

In the European League against Rheumatism (EULAR) recommendations for the management of RA from 2019, there is no information about monitoring for the side effects of therapy or how to manage them. The authors of those recommendations assumed that the clinicians prescribing the drugs would be aware of any potential toxicity [1]. In addition, the reader is directed to a series of documents with information about the safety of the medications. None of them, however, address the problem of hepatotoxicity. In the American College of Rheumatology (ACR) recommendations, only the aminotransferase level is proposed for monitoring for eventual liver injury [2]. Precise guidelines for monitoring patients undergoing methotrexate therapy comprise recommendations from the Joint American Academy of Dermatology and European Academy of Dermatology and Venerology for the treatment of psoriasis vulgaris [5, 6].

While liver biopsy is a common method for assessing liver damage, the procedure is connected with a considerable risk of pain, bleeding and other complications. Therefore, to determine the level of liver fibrosis while avoiding the aforementioned complications, as recognized alternatives, the Enhanced Liver Fibrosis (ELF) test and transient elastography (TE) were performed in our study.

The ELF test is a simple blood test used to assess liver fibrosis. It was applied in our patients along with other routine blood tests. The ELF panel includes three serum biomarkers of extracellular matrix (ECM) fibrogenesis and fibrous protein degradation: hyaluronic acid (HA), procollagen III N-terminal peptide (PIIINP) and tissue inhibitor of matrix metalloproteinase 1 (TIMP1). The three included biomarkers are combined in an algorithm for calculation of the ELF score, which correlates to the liver fibrosis level found on liver biopsy [7]. In addition, serial measurement of the PIIINP level in blood was introduced to the guidelines for treating psoriasis with methotrexate instead of elective liver biopsy after reaching a cumulative MTX dose of 3500–4000 mg [5, 6].

TE is an imaging technique used to assess liver fibrosis by measuring the propagation of low-frequency ultrasound (50 Hz). The velocity of ultrasound waves is determined by the stiffness of the tissue, with faster propagation in harder tissues. TE is a quick test and can be done repeatedly. American elastography guidelines for the treatment of psoriasis recommend using TE as the standard method for assessing MTX-induced liver toxicity [5]. It is also a core diagnostic procedure to exclude liver cirrhosis in viral hepatitis [8, 9].

## Objectives

The aim of this research was to evaluate the usefulness of noninvasive methods of liver examination, acknowledged in assessing liver fibrosis in viral hepatitis or psoriasis, including transient ultrasonography (TE) and determination of serum markers (PIIINP, ELF score), in patients with RA receiving methotrexate.

## Patients and methods

### Case definition

It is a retrospective study, focusing on non-invasive diagnostic procedures acknowledged in diagnosing liver fibrosis in diseases, such as viral hepatitis or psoriasis.

### Data collection and participants

We recruited 96 patients (72 women and 24 men), all of whom were patients of Rheumatology Clinic associated with Department and Clinic of Rheumatology in Wroclaw University Hospital. In the study we analyzed data regarding patients biometrics, methotrexate treatment and its effect on treated patients. Initial data was collected from medical history and completed on the recruitment visit. During that visit in a short questionnaire, we collected lacking physical data from all patients and information regarding the treatment schedule, as follows: age; weight; body mass index (BMI); duration of methotrexate therapy; cumulative dose of methotrexate; concomitant therapy; comorbidities, and alcohol intake. During the physical examination, the number of painful and swollen joints was counted. On the basis of the aforementioned parameters and Patients Global Health scale, RA activity was evaluated using the Disease Activity Score for 28 joints (DAS-28) scale. Statistical data of the examined patients are presented in Table 1. Blood samples were collected for routine laboratory tests: complete blood count; alanine aminotransferase (ALT); aspargine aminotransferase (AST); c-reactive protein (CRP), erythrocyte sedimentation rate (ESR), creatinine. The PIIINP, HA and TIMP1 levels were measured in 10 ml of blood collected in EDTA vials. Blood samples were centrifuged immediately after donation at 1500 g for 10 min, and then serum was collected to the vials. The concentrations of PIIINP, HA and TIMP1 in serum were measured using the ADVIA Centaur Enhanced Liver Fibrosis test.

**Table 1:** Statistical data of patients recruited to study (constitutional parameters, data regarding methotrexate therapy and laboratory findings)

	Number of patients	Min	Max	Median	Mean	SD	Reference range
Age (years)	96	19	85	60.5	59.85	12.13	
Weight (kg)	96	46	140	70	73.91	16.66	
BMI (kg/m^2^)	96	16.1	46.62	26.2	26.91	4.92	< 18.5 underweight 18.5– < 25 healthy weight 25– < 30 overweight 30 and above obesity
MTX dose per week (mg)	96	10	30	17.5	16.54	7.37	
Duration of MTX treatment (years)	96	0.1	21	4	6.41	11.18	
Cumulative dose of MTX (mg)	96	12.5	27,300	3140	4775.37	5123.33	
DAS 28	96	0.98	8.42	4.38	4.5	1.65	< 2.6 remission 2.6–3.2 low disease activity > 3.2– ≤ 5.1 moderate disease activity > 5.1 high disease activity
PIIINP (ng/ml)	96	2.45	25.37	7.3	8.52	3.96	1.7–4.2
ELF-1	82	7.2	12.53	9.49	9.67	1.1	< 7.7 low risk of liver fibrosis 7.7–9.8 intermediate risk of liver fibrosis > 9.8 high risk of liver fibrosis
Hb (g/dl)	96	9	18	13.2	13.26	1.45	Female 12–16 Male 14–18
ALT (U/l)	96	7	196	21	27.25	23.28	35
AST (U/l)	96	11	122	24.5	27.55	14.64	35

*SD* standard deviation, *BMI* body mass index, *MTX* methotrexate, *DAS 28* Disease Activity Score of 28 joints, *PIIINP* procollagen III N-terminal peptide, *ELF-1* Enhanced Liver Fibrosis score, *Hb* hemoglobin, *ALT* alanine aminotransferase, *AST* aspargine aminotransferase

Transient elastography examinations were all performed with a Siemens Acuson S2000 by the same examinator (MG). For examination, the patient was in a supine position with the right upper limb under the head. Measurements were taken from the right lobe of the liver through the intercostal space. The results were measured in kilopascals. The distinguishing point of liver fibrosis was established at 7.1 kPa and 13 kPa for liver cirrhosis which corresponds with METAVIR F2 and F4 degrees in vast majority of analyzed studies [8, 10–14]. In case of acquiring fewer than 10 or lesser than 60% valid shots or the variability of measurements was higher than 30% of the median value of liver stiffness the result was counted as unreliable (equivocal result).

For further analysis of transient elastography result patients were divided into two groups according to whether they had risk factors for liver damage. We selected diabetes mellitus (DM), obesity (BMI over 30) and preexisting liver disease (non-alcoholic fatty liver disease diagnosed before recruitment visit, based on abdomen ultrasonography) as the most important risk factors. Having at least one risk factor was the criterion to be selected for the study group, while the control group comprised patients with no risk factors. Statistical data of patients in these groups are presented in Table 2.

**Table 2:** Transient elastography results divided into groups depending on the presence of liver fibrosis risk factors

Group	1	2	Total
Number of patients	42	41	83
Elastography result			
Minimum	0	0	0
Maximum	4	4	4
Mean	2.09	1.29	1.68
Standard deviation	1.58	1.43	1.55
Variance	2.51	2.04	2.4
Relative risk (RR)			
RR	Reference	2.103	
95% CI		1.278–3.458	
*p* value (Chi-squared test)		0.001	
Odds ratio (OR)			
OR	Reference	4.308	
95% CI		1.719–10.798	
*p* value (Chi squared test)		0.001	

1—Study group (with liver fibrosis risk factors), 2—Control group (without risk factors)

### Inclusion and exclusion criteria

The selection criteria were as follows: age over 18 years; confirmed diagnosis of rheumatoid arthritis; ongoing treatment with methotrexate; and signed written consent. Active viral hepatitis, alcohole abuse (> 7 units per week) and malignant neoplastic disease disqualified patients from participation.

### Statistical analysis

Statistical analysis was performed using Statistica ver. 10.0. Pearson’s correlation coefficient was used to evaluate linear correlations and Spearman’s correlation coefficient was performed to assess non-linear interconnections. Mann–Whitney *U* test was used in nonparametric statistics. A multivariate linear regression was performed to distinguish the parameters defining the ELF-1 and PIIINP scores. In the model, we used variables that showed a linear correlation with the assessed laboratory markers of fibrosis: for both parameters, we used MTX treatment time, cumulative MTX dose and the DAS28 result; in addition, we analyzed body weight for PIIINP and age for ELF-1.

## Results

### Transient elastography

Ninety-one patients underwent transient elastography examination. Eight of them had equivocal results. Only 23 patients (27.7%) had no signs of fibrosis on the performed scans, and 17 (20.5%) had a result suggesting liver cirrhosis. Due to the large group of participants with the TE result of 13 kPa and above, its statistical evaluation was performed. Out of 17 patients 12 (70.6%) were women, 7 (41.2%) had BMI above 30, 11 (64.7%) had concomitant therapy with other hepatotoxic drug with 5 (29.4%) taking statins, 9 (2.9%) had preexisting liver disease and 5 (29.4%) had DM. Mean age of that group was 62.53, mean cumulative MTX dose was 5679.4 mg. The TE result correlated only with the BMI (Spearman’s correlation coefficient, 0.23). More obese patients tended to have more advanced liver fibrosis. There was no correlation of the TE result with any other parameter assessed in our study: PIIINP serum level (*p* = 0.5); ELF score (*p* = 0.56); cumulative MTX dose (*p* = 0.33). No other common risk factors for liver fibrosis correlated with the TE result: DM (Mann–Whitney *U* test, *p* = 0.86); concomitant therapy with hepatotoxic drugs (Chi-squared test, *p* = 0.42).

**Fig. 1:**
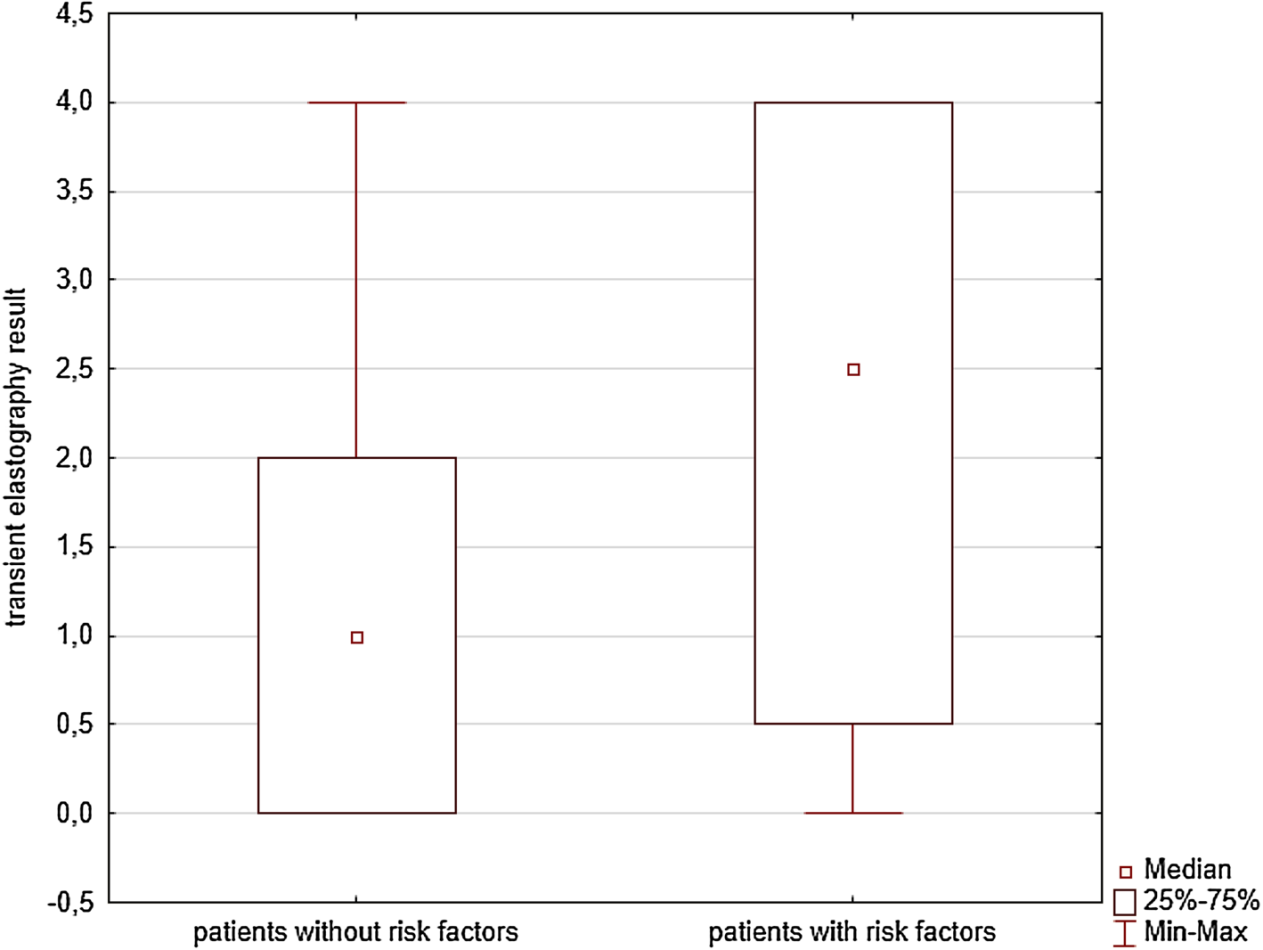
Transient elastography result depending on the presence of risk factors (Statistica ver. 10.0)

Eighty-three patients with unequivocal results were divided into two groups depending on whether they had risk factors for liver injury (DM, obesity, preexisting liver disease). The study group consisted of 42 patients, and the control group consisted of 41 patients. There was a statistically significant difference between the two groups (Fig. 1), with higher liver fibrosis in the prior group (TE result >  = 7.1 kPa 28/42 vs 13/41, HR 2.103, Mann–Whitney *U* test, approximately 0.02). In both groups, there was no correlation between the TE result and the cumulative dose of MTX (Mann–Whitney *U* test, *p* nearly 0.97 for the study group and approximately 0.34 for the control group).

### Procollagen III N-terminal peptide serum level

The PIIINP serum level was measured in 96 patients receiving MTX. Forty-three of them (44.8%) had elevated values. There was a weak positive but statistically important correlation between the PIIINP level and body weight (*r* = 0.23; *p* = 0.028), duration of MTX therapy (*r* = 0.2; *p* = 0.05), cumulative dose of methotrexate (*r* = 0.28; *p* = 0.007) and RA activity assessed by the DAS-28 score (*r* = 0.28; *p* = 0.028). The mean PIIINP level measured in blood samples from patients weighing less than 80 kg was 7.76, and that measured in blood samples from patients weighing over 80 kg was 10.33. For further analysis, the cumulative dose threshold was set at 4000 mg, which corresponds with the recommendation for performing elective liver biopsy. The mean PIIINP level in the serum of patients with a cumulative dose less than 4000 mg was 7.86 ng/ml, and that in patients with higher MTX intake was 9.72 ng/ml. Patients with low RA activity (DAS-28 score < 3.2) had a mean PIIINP level of 7.61, and those with high RA activity (DAS-28 score > 5.1) had a PIIINP level of 11.32 ng/ml. DM was an independent risk factor for higher PIIINP levels (*p* = 0.001). In diabetic patients, the median PIIINP level was 11.38 ng/ml, compared with 7.15 in nondiabetic patients. To better define the PIIINP result a multivariate linear regression was performed. In the initial model we analyzed all aforementioned parameters and after subsequent removal of irrelevant variables we acquired statistically important model (*R*^2^ = 0.177; *p* < 0.003) with DAS 28 (*p* = 0.009) and MTX treatment duration (*p* = 0.012) as describing parameters.

### Enhanced liver fibrosis score

The ELF score was determined in 82 patients in our study. Only two of them were at a low risk of hepatic fibrosis, with ELF scores < 7.7, and 33 patients were at a high risk, with ELF scores > 9.8. There was a weak, positive but statistically significant correlation between the enhanced liver fibrosis score and parameters, such as age (*r* = 0.38; *p* < 0.001), duration of MTX treatment (*r* = 0.2991; *p* = 0.007) and cumulative dose of methotrexate (*r* = 0.30; *p* = 0.007). RA activity had strong, positive interconnection to ELF-1 score value (*r* = 0.51; *p* < 0.001). The mean ELF score in patients older than 65 was 10.35, and that in patients younger than 65 was 9.41. The same threshold for the cumulative dose of methotrexate as in the analysis of the PIIINP level was assumed in the analysis of the ELF score. The mean ELF score in patients with cumulative doses below 4000 mg was 9.51, while it was 9.85 in patients with cumulative doses over 4000 mg. However, a greater difference was seen with greater cumulative doses of methotrexate (i.e., when we used a threshold of 6500 mg, which corresponds to 5 years of 25 mg of MTX intake per week, the result was 9.39 in patients who did not meet the threshold vs 10.28 in patients who exceeded the threshold). The most relevant correlation was observed between RA activity and the ELF score. In patients with low RA activity, the mean ELF score value was 8.95, and in patients with high RA activity, it was 10.55. Correlation of both PIIINP and ELF-1 with examined parameters is presented in Table 3. A multivariate linear regression of parameters listed above was performed. After gradual removal of irrelevant variables we received statistically important model (*R*^2^ = 0.465; *p* < 0.001) with age (*p* = 0.002), MTX treatment duration (*p* = 0.013) and DAS28 (*p* < 0.001) as describing parameters.

**Table 3:** Correlation of laboratory fibrosis markers (PIIINP, ELF) and examined parameters (age, body weight, BMI, MTX dose, cumulative MTX dose, duration of MTX treatment, RA activity)

	Age	Weight	BMI	MTX dose per week	Duration of MTX treatment	Cumulative dose of MTX	DAS 28
PIIINP							
Pearson correlation coefficient	*r* = 0.1473	*r* = 0.2301	*r* = 0.1899	*r* = 0.0598	*r* = 0.2017	*r* = 0.2774	*r* = 0.2752
Test result	*p* = 0.152	*p* = 0.028	*p* = 0.071	*p* = 0.562	*p* = 0.05	*p* = 0.007	*p* = 0.028
ELF-1							
Pearson correlation coefficient	*r* = 0.3853	*r* = 0.1145	*r* = 0.0883	*r* = 0.0321	*r* = 0.2991	*r* = 0.3014	*r* = 0.51
Test result	*p* < 0.001	*p* = 0.318	*p* = 0.442	*p* = 0.775	*p* = 0.007	*p* = 0.007	*p* < 0.001

## Discussion

Because of its efficacy and low cost, methotrexate is and probably will continue to be used as the standard therapy for RA [1, 2]. The long-term administration of MTX requires regular monitoring for side effects, such as hepatotoxicity. However, due to a lack of precise recommendations, most physicians only monitor the aminotransferase level in serum, which may but does not always correspond to the extent of liver damage. Liver fibrosis might progress silently without an apparent elevation of the aminotransferase serum level [11, 12, 15, 16]. Liver biopsy, on the other hand, carries the risk of morbidity and even mortality, which highlights the need for new, less invasive methods.

Transient elastography, as a noninvasive method used to measure liver stiffness, is broadly used to assess liver fibrosis, especially in patients with viral hepatitis [8]. New guidelines regarding the treatment of psoriasis also include TE examination in the evaluation of patients for MTX hepatotoxicity. In various studies, it has been compared to liver biopsy, with relatively good specificity and sensitivity for detecting fibrosis *F* ≥ 2 on the METAVIR scale. TE is less precise in discriminating lower stages of liver fibrosis (F0–F1) [8, 9]. In our study, we did not find a correlation between the cumulative dose or the duration of therapy and the transient elastography result. However, the correlation between the TE result and the BMI of the patient was statistically significant. Our results correspond with those of a number of studies and might be associated with the idea that just administering methotrexate does not lead to liver fibrosis and that other factors are required [3, 12, 14, 17]. MTX might accelerate the process of fibrosis in predisposed patients, especially those with metabolic syndrome. Studies comparing RA and psoriasis or psoriatic arthritis have demonstrated that liver fibrosis is more common in patients with the latter disease [4]. Patients with psoriatic arthritis are also at a higher risk of developing metabolic syndrome.

In most studies, an interconnection between the cumulative dose of MTX and the TE result was not observed [3, 10, 12, 14–20]. Only two studies reported the opposite result [11, 13]. Arena et al. assessed the correlation between the cumulative MTX dose and TE result in 100 patients without any risk factors for liver injury. The disqualification criteria included DM, metabolic syndrome, alcohol abuse, concomitant therapy with hepatotoxic drugs and relevant increase in aminotransferase during therapy [11]. The main disadvantage of this study was that the study group was not compared with patients with risk factors. In our study, such a comparison was performed; however, there was no correlation between the TE result and the cumulative MTX dose in either group. Our control group differed from that in the previous study in that we did not disqualify patients using other hepatotoxic drugs (there was no correlation between concomitant hepatotoxic therapy and the TE result in our study). Lertnawapan et al. conducted a study of 108 participants with RA. The disqualification criteria were chronic liver disease, elevated liver enzymes (twice the upper limit or more), alcohol abuse, concurrent therapy with hepatotoxic drugs, and chronic heart or kidney disease. Statistical analysis in this study indicated a positive correlation between liver stiffness and BMI, fatty liver, ALT and cumulative MTX dose [13]. As in the previous study, there was no comparison with patients with risk factors. In addition, data regarding disqualification criteria were not consistent; initially, there was information indicating that patients taking other hepatotoxic drugs were not recruited to this study, but in the results, a correlation between liver stiffness and statin intake was observed (statins are potentially hepatotoxic medicines) [21].

PIIINP is a coproduct of collagen fiber metabolism that may reflect the intensity of the process of fibrosis in humans. In a number of publications, serum PIIINP measurements were utilized to assess the liver toxicity of methotrexate in psoriatic patients [22–28]; on the other hand, there have only been a few studies examining patients with RA [28].

There was a significant linear correlation between the PIIINP level and cumulative methotrexate dose in our study, which is in agreement with the results of other studies [22, 24]. There was only one publication in which such a relation was not observed. Chladek et al. recruited 49 patients with psoriasis, who were divided into three groups regarding actual therapy. The first group comprised MTX-naïve patients (who used only topical treatment before the study) who just started treatment with methotrexate; the second group comprised patients treated with biological therapy (MTX therapy discontinued at least 1 year before the study); and the third group comprised patients undergoing ongoing treatment with MTX lasting more than 2 years. There were no statistically significant differences in the PIIINP serum level among the groups; however, this might be the result of the very small sample size (24 vs 15 vs 10) [25]. On the other hand, after performing multivariate linear regression a statistically important model has been built in which MTX cumulative dose was irrelevant. Similar observations were made by van der Voort et al. [28].

Rheumatoid arthritis activity measured by the DAS-28 scale was an important risk factor for an elevated PIIINP serum level in our study. This observation corresponds with the results of other studies concerning RA as well as psoriasis [23, 24, 26, 28]. Psoriatic arthritis is a common reason for an elevated serum PIIINP level. There has only been one study in which such a correlation was not confirmed; however, it might have been caused by the small number of patients with an elevated PIIINP serum level (only 16 patients) [27]. Active arthritis as a process involving increased collagen turnover, which may overestimate the results. To minimize the risk of false readings, a series of PIIINP measurements instead of one should be performed. However, among psoriasis, psoriatic arthritis and rheumatoid arthritis, the latter is characterized by the highest mean PIIINP level [28].

Because PIIINP is one of three biomarkers that are used to calculate the enhanced liver fibrosis score, there is a straight correlation between them. However, the ELF score is regarded as more specific to liver fibrosis [28]. Nevertheless, a different observation was made in our research, with a strong positive correlation with rheumatoid arthritis activity. Lynch et al. conducted a study assessing both the TE result and PIIINP level, finding no correlation [14]. A similar observation, including the ELF score, was made in our study.

The main limitations of our study are to small examined group to determine strong, unequivocal conclusions and the lack of histopathological verification of study results, as we performed only noninvasive procedures. However, the evaluated diagnostic procedures are recognized in screening for liver fibrosis in viral hepatitis, NAFLD, and psoriasis, with AUC/ROC calculations measured and confirmed to be significant [8, 10]. In this regard, the results and conclusions should be interpreted with caution.

## Conclusions

Methotrexate plays a fundamental role in RA treatment and requires proper monitoring for side effects of therapy. Liver biopsy carries a potential risk, which is why new methods for hepatotoxicity assessment are needed. Serum biomarkers, such as the PIIINP level and ELF score, may be analyzed in standard blood samples; however, they are not organ specific, and readings may vary depending on RA activity. Another disadvantage is that it presents the actual process of fibrogenesis, and the results may be negative, even in end-stage cirrhosis. Transient elastography is an organ-specific examination that if performed by a skilled ultrasonographer might provide useful information regarding the actual liver condition. This examination technique is acknowledged as a standard procedure in monitoring for MTX-induced hepatotoxicity in psoriatic patients or assessing the liver condition in patients with viral hepatitis. In spite of aforementioned restrictions of both methods it is necessary to highlight their usefulness in screening of liver damage, especially transient elastography.

## Supplementary Information

Below is the link to the electronic supplementary material.

Supplementary file1 (DOCX 15 KB)

## Data Availability

The data sets generated and analysed during the current study are available from the corresponding author on reasonable request.
