# Supplementary material for: Usefulness of noninvasive diagnostic procedures for assessment of methotrexate hepatotoxicity in patients with rheumatoid arthritis
Source: Rheumatol Int. 2021 Dec 6;42(4):631–8. doi: 10.1007/s00296-021-05059-z (PMC8940880; doi:10.1007/s00296-021-05059-z)
Supplement: Supplementary file 1 — Supplementary file1 (DOCX 15 KB) [file 296_2021_5059_MOESM1_ESM.docx]

Data of related congress abstract publication:

1. Swierkot J, Frankowski M, Skoczynska M, et al (2017) THU0194 The role of enhanced liver fibrosis (ELF) score in patients with rheumatoid arthritis treated with methotrexate. 276.1-276. https://doi.org/10.1136/annrheumdis-2017-eular.6865
